# Simulation Studies of Dynamical Heterogeneity in a Dense Two-Dimensional Dimer–Solvent System with Obstacles

**DOI:** 10.3390/e26121086

**Published:** 2024-12-12

**Authors:** Piotr Polanowski, Andrzej Sikorski

**Affiliations:** 1 Department of Molecular Physics, Faculty of Chemistry, Lodz University of Technology, Zeromskiego 116, 90-543 Lodz, Poland; 2 Faculty of Chemistry, University of Warsaw, Pasteura 1, 02-093 Warsaw, Poland; sikorski@chem.uw.edu.pl

**Keywords:** anomalous diffusion, dynamic lattice liquid, lattice model, molecular crowding, Monte Carlo method

## Abstract

A coarse-grained model of a two-dimensional colloidal suspension was designed. The model was athermal and, in addition, a lattice approximation was introduced. It consisted of solvent (monomer) molecules, dimer molecules, and immobile impenetrable obstacles that introduced additional heterogeneity into the system. Dynamic properties were determined by a Monte Carlo simulation using the dynamic lattice liquid simulation algorithm. It is shown that there is a range of obstacle concentrations in which different diffusion characteristics were observed for dimers and solvents. In the system studied, it is possible to define the ranges of concentrations of individual components (solvent, dimers, and obstacles), in which the nature of the movement of dimers and solvents is different (normal diffusion vs. subdiffusion). The ratio of diffusion coefficients of solvent molecules and dimers for short times does not depend on the concentration of obstacles, while for long times, the ratio increases but remains independent of the concentration of the dimer.

## 1. Introduction

The dynamic behavior of colloidal suspensions is important to many technologies in various fields, including ceramics, food processing, or pharmaceuticals. In colloidal dispersions, colloidal dynamics are much more complex compared to ordinary solutions. The motion of an object induces a flow field in the solvent, which affects neighboring colloid particles, so it is important to include the solvent in the considerations, which is referred to as hydrodynamic interactions. The wide range of time and length scales, the difference in size and mass between the colloid and the solvent particles, and complex interactions in such systems make the dynamics of colloids far from being completely understood. Experimental studies of the dynamics of anisotropic colloids have mostly been carried out only at low particle concentrations [1,2,3,4,5,6]. Such two-dimensional and quasi-two-dimensional systems have been studied experimentally [7,8,9] and simulated using molecular dynamics and Monte Carlo methods [7,10,11].

Thus, in colloidal systems, it is very important to understand the microscopic sources of molecular transport. Most often, we describe such processes using the concept of diffusion, and the diffusion coefficient is defined as *D* = <Δ*r*^2^>/2*dt*, where <Δ*r*^2^> is the mean square displacement (MSD) of a moving object, *d* is the dimension of space, and *t* is time; the determination of the diffusion coefficient is possible if MSD~*t*. In disordered systems, the behavior of the MSD is more complex; in addition to regions where it depends linearly on time, we also have regions of nonlinear dependence, where the MSD on time: MSD~*t*^α^ with α < 1 (subdiffusion). Such systems are studied by theoretical methods such as continuous time random walk (CTRW) [12,13,14,15,16], “fractional Brownian motion” [17,18], and obstructed diffusion [7,19,20,21]. However, despite both numerous experiments [17,22,23,24,25,26,27,28,29,30,31,32,33,34,35,36,37] and computer simulations [17,38,39,40,41,42,43,44,45,46,47,48,49,50,51,52,53], the dynamics in such systems are still far from being understood. Impediments to the motion of liquid molecules can be caused not only by immobile obstacles but also by objects with different mobility than the liquid. Most often, we have to deal with a matrix of moving obstacles whose mobility is much lower. For such systems, subdiffusive motion is observed at intermediate periods, but there is always a return to Fickian diffusion. Therefore, in such systems, it is not possible to talk about the phenomenon of classical percolation—even above the static percolation threshold, we are dealing with the unrestricted movement of liquid molecules [54,55,56,57]. The second important factor, affecting the parameters of anomalous diffusion, is the difference in the size of liquid particles and obstacles. It is most often assumed that large objects move slow enough so their motion can be ignored if one is only interested in the dynamics of small objects [58,59,60]. The influence of interactions should also be noted [41,61,62,63], though we will not address this further here. It should be added, however, that the high density of colloidal systems means that the motion of individual objects depends on the motion of elements in their vicinity, so the significant influence of cooperative processes such as hydrodynamics must be taken into account [7,8,11,12,64]. To perform the simulation, an algorithm is needed that is able to efficiently sample configurations for very dense systems. At the same time, when considering the motion of a given object, the influence of other stationary and moving objects must be taken into account, i.e., the effect of the correlations mentioned above.

A large number of papers have been published about diffusion in crowded environments in two and three dimensions [57,58,61,65,66,67]. Most of these works study the movement of non-interacting objects (single agent models or Lorentz gas). In the dynamic lattice liquid (DLL) model we used, we have a completely different model of transport and at the same time, an opportunity to study dense systems at very long times. In the DLL model, cooperative motion loops are responsible for hydrodynamic interactions. Their characteristics are thoroughly presented and demonstrated in Ref. [64]. This model was applied to a simple model of a colloidal dispersion, where cooperativity in motion is crucial. Systems of this type, i.e., containing two types of moving elements of different sizes and shapes containing obstacles, have not yet been studied. Therefore, we designed a coarse-grained lattice model. We placed solvent molecules, dimer molecules, and impenetrable and immobile obstacles in the system under study. The properties of such systems were determined using Monte Carlo simulations. Due to the high densities, a suitable simulation algorithm had to be used. Thus, the DLL model, based on the concept of cooperative movements, was chosen [65].

## 2. Materials and Methods

In order to study the proposed system, a coarse-grained lattice model was designed. Both approximations were designed to reduce the configuration space and simplify the interactions and to significantly speed up the calculations. In the DLL model, there is only one object at each site of a two-dimensional triangular lattice; in our case, it is a solvent molecule, a half of a dimer, or an impenetrable obstacle [68,69,70]. As in the liquid models, it was assumed that the dense system has some excess volume so each molecule can vibrate around its position, and this average position was defined by the lattice site in which it was placed. Objects are not in a position to move for longer distances from time to time, because the entire neighborhood (all neighboring sites of the lattice) remains occupied. Random translations of objects over distances exceeding the vibrational range, i.e., from an average position (a given lattice site) to a neighboring location in the lattice, are only possible in a cooperative manner. This is how the DLL algorithm works, where cooperative rearrangements take the form of closed displacement loops involving at least three sites of the lattice. In addition to enabling the study of systems with the highest density, the DLL model satisfies the continuity equation and provides correlated molecule motions, similar to what occurs in a real fluid. The dynamic properties generated by this model have been shown to be fully consistent with those in real liquids [68]. It has also been shown that DLL is able to correctly reproduce dynamics not only in liquids but also in other complex physical systems, for example, reaction diffusion front analysis [69], the structure and dynamics of dense polymer solutions [70], the kinetics of polymerization and copolymerization processes [71,72], and dynamics in disordered systems [57,59,61,73]. The models used in the latter studies became the starting point for the design of the present study.

**Figure 1:**
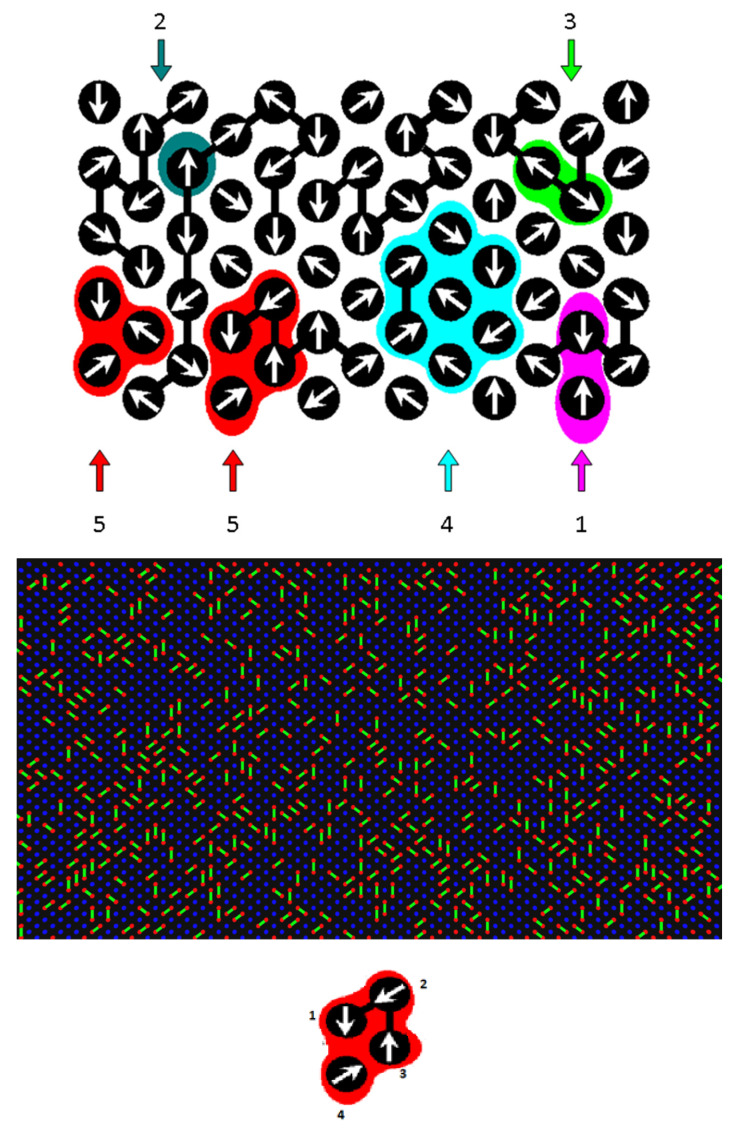
**Upper Panel**: an illustration of a vector field representing the attempts of molecules to move toward neighboring sites of the lattice in the DLL model. The following situations are distinguished: (1) elements try to exchange places in the lattice (unsuccessful attempt), (2) movement occurs from a point to which no other element tries to reach (unsuccessful attempt), (3) movement leads to breaking a bond in the dimer (unsuccessful attempt), (4) a solvent molecule jumps over a dimer bond (unsuccessful attempt), and (5) each element exchanges position with its neighbor—a cooperative movement loop (successful attempt). **Middle Panel**: two-dimensional system consisting of dimers in an explicit solvent. The concentration of the dimer is 0.5. **Lower Panel** shows the motion in a cooperative motion loop of elements linked by bonds (the variant 5 from the upper panel of the figure is shown; one can see that in this case, the arrows assigned to the objects form a closed loop). As a result of the movement indicated by the arrows, element 3 will swap position with element 2, element 2 with element 1, and element 4 with element 3, while the bond will not break.

A two-dimensional system embedded on a triangular lattice with periodic boundary conditions imposed along all axes of the coordinate system was investigated. The disordered system was modeled by introducing immobile and impenetrable obstacles. Each solvent molecule and obstacle occupied one site of the lattice, so both were the same size. In contrast, a dimer consisted of two elements (mers) connected by a rigid bond; these mers were the same size as the solvent and obstacle. These obstacles were randomly generated with an assumed concentration. If a lattice site was an obstacle, it did not participate in any cooperative loop. The mentioned objects occupied all lattice sites of the system. The concentration of obstacles in the system *c* was defined as the ratio of the number of lattice sites occupied by obstacles to the total number of lattice sites in the Monte Carlo box: *c* = n/*L*^2^, where *n* is the number of obstacles in the system. The concentration of dimers was calculated as *d* = 2*m*/*L*^2^, where *m* was the number of dimers in the system. The system was athermal, that is, because of the assumption that all long-range interactions of the objects are the same (good solvent), these interactions could be ignored. Thus, the only potential interaction in the model was the excluded volume of objects realized by the prohibition of the double occupation of lattice sites. The Monte Carlo simulations were carried out employing the DLL algorithm in order to determine the properties of this system. A scheme of this algorithm is shown in Figure 1 (upper panel). At a given pseudo-time unit (a Monte Carlo step), the following actions were performed: each location (a lattice site) was assigned a unit vector with a random direction pointing to one of the 6 nearest neighboring sites of the lattice (Figure 1, upper panel). Each vector represented the direction along which an object was then attempted to move to a neighboring location, i.e., to a neighboring lattice site. All closed position exchange loops in the system, consisting of at least three elements and formed by these vectors, were identified. These loops are marked in Figure 1 (upper panel) with different colors. The remaining objects, i.e., those whose vectors did not form a closed loop, were immobile at a given time step. Closed loops indicated pathways for possible cooperative rearrangements of objects by moving objects to neighboring locations (each object replacing its neighbor). Displacements along some loops cannot be performed if the movement occurs from a point to which no other element tries to reach, the movement leads to breaking a bond in the dimer (unsuccessful attempt), or a solvent molecule jumps over a dimer bond. The unit of time (a Monte Carlo step) was assumed to be a discrete variable, in which the positions of all particles were attempted to change simultaneously in all cooperative movement loops existing in the system at a given step. Simulations were performed in an *L* × *L* Monte Carlo box, where *L* = 256. It was shown that in the DLL model for systems in boxes with edges *L* > 64, the statistics of cooperative motion loops do not depend on the size of the box [17]. More than 40,000 trajectories were generated for each random obstacle matrix, and each trajectory took 10^9^ Monte Carlo steps. The simulations were repeated about 30 times, each simulation run was performed for a different obstacle matrix, and the results were averaged over all runs for a given set of concentrations *c* and *d*. Simulations were carried out for three systems with the concentration of dimers at 0.10, 0.50, and 0.70 and with obstacle concentration varying from 0 to 0.36. The rest of the system was filled with solvent molecules.

## 3. Results and Discussion

In order to gain basic knowledge about the dynamics of the considered systems, first we analyze the dynamic behavior of the systems without the presence of obstacles that can be treated as a state of reference. The basic quantity used to obtain such characteristics for dimers and solvent molecules is a diffusion coefficient which connects the mean square displacement (MSD) with time using the Einstein relation in two dimensions as follows:

(1) $$\langle {∆r(t)}^{2}\rangle =4Dt$$

where <Δ*r*^2^(*t*)> is the MSD of the movable objects (liquid molecules). The MSD is defined as

(2) $$\langle {∆r(t)}^{2}\rangle =\frac{1}{n}\sum_{i=1}^{n}{\left[r_{i}(t)-r_{i}(0)\right]}^{2}$$

where ***r****_i_*(*t*) denotes the space coordinates of the ith bead at time *t* and *n* is the number of movable elements. In the case of the normal (Fickian) diffusion, according to Equation (1), the MSD is proportional to the time *t*. In disordered systems, this law is not always fulfilled, and the anomalous diffusion can appear [13].

(3) $$\langle {∆r(t)}^{2}\rangle \;\sim \;t^{\alpha }\;\sim \;t^{2/d_{w}}$$

with α = 2/*d_w_* as the anomalous diffusion exponent. The diffusion is hindered in conditions with α ≤ 1 or *d_w_* ≥ 2.

The other quantity, which allows us to monitor the dynamics of dimers, is the autocorrelation function of the end-to-end vector of dimer ***R***.

(4) $$\cos\left(\varphi \right)=\frac{1}{n}\sum_{i=1}^{n}\frac{R_{i}\left(0\right)R_{i}(t)}{R_{i}^{2}(0)}$$

where ***R***(0) and ***R***(*t*) concern time *t* = 0 and *t*, respectively. This quantity allows for tracking the rotational movement of dimers.

**Figure 2:**
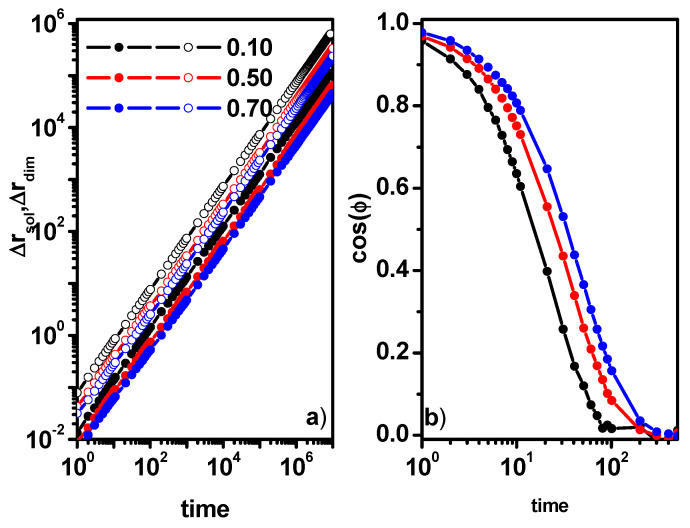
The MSD of the solvent (open symbols) and dimer center of mass (solid symbols) (**a**) and the end-to-end vector of the dimer autocorrelation function (**b**). Concentrations of dimers are given in the inset.

Figure 2a shows the MSD of solvent molecules and of dimers in the cases considered (throughout this work, we mean the diffusion of the center of mass of a dimer). The mobility of the solvent is obviously greater than that of the dimer. As expected, with increasing dimer concentration, the mobility of both solvent molecules and dimers decreases. This means that the less mobile dimers present a significant obstacle to the solvent molecules. Figure 2b shows the end-to-end autocorrelation function calculated according to Equation (4). It can be observed that with increasing dimer concentration, the relaxation time also increases. One can also see that dimers quickly forget their original size (relaxation times are of the order of 10^2^ for all dimer concentrations considered). In order to further characterize the solvent behavior, in addition to the characteristics described by Equation (2), one can use the position autocorrelation function (PAF) of the solvent. This function is defined as

(5) $$\rho \left(t\right)=\frac{1}{n}\sum_{i=1}^{n}m_{i}\left(0\right)m_{i}\left(t\right)$$

where *m_i_*(*t*) = 1 or 0, depending on if the ith object occupies or does not occupy its original position after time *t*, respectively. This function provides us with information whether or not the ith object occupied its original position (at *t* = 0) and at a given time *t*, respectively.

**Figure 3:**
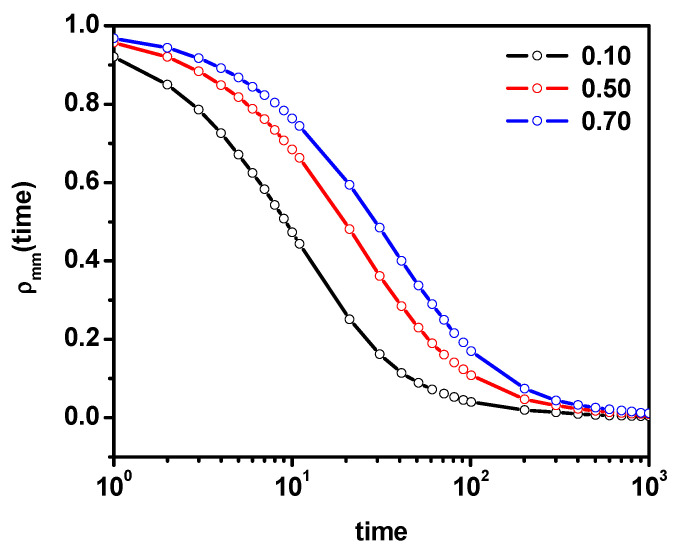
The solvent position autocorrelation function ρ(t) for various concentrations of dimers.

In Figure 3, we plotted the position autocorrelation function of solvent molecules for some dimer concentrations. As expected, with increasing dimer concentration, the position relaxation time increases. The above characteristics obtained for systems without obstacles gives a clear and coherent picture of the dynamic behavior of solvent and dimer mixtures related to changes in hydrodynamic interactions with an increasing concentration of dimers. Generally, in all cases, we do not observe any deviations from Einstein diffusion, and the changes in dynamics are related to the change in dimer concentration. The question arises whether the above picture will be changed as a result of introducing obstacles in the form of stationary impenetrable points. For this purpose, we consider an intermediate system in which the dimer concentration is 0.5 and solvent molecules will be systematically replaced by obstacles.

**Figure 4:**
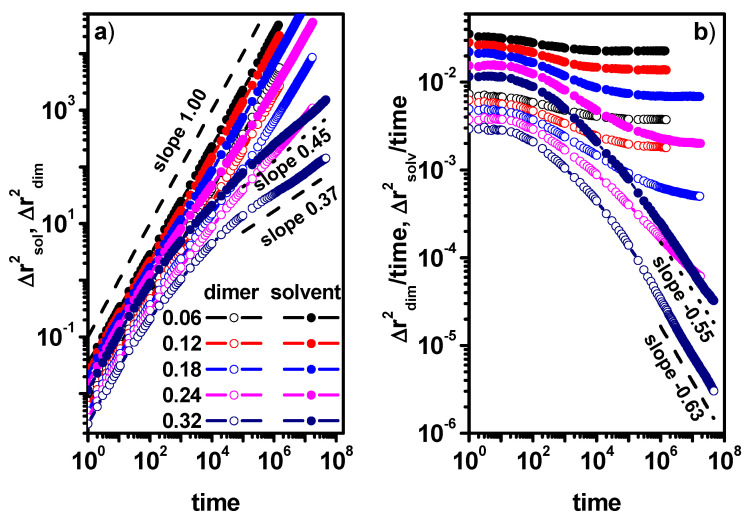
The MSD for the solvent (solid symbols) and dimers (open symbols) as a function of time (**a**) and MSD/time as a function of time (**b**). The concentrations of obstacles are given in the inset. The concentration of dimers is 0.5.

Figure 4a shows the MSD as a function of time for solvent molecules and dimers for various concentrations of obstacles. Because the conventional plot MSD vs. time shown in Figure 4a does not clearly show the changes in the diffusion character, we also plotted MSD/time, which is shown in Figure 4b. In MSD curves for both solvent molecules and dimers, we can distinguish three typical regions. The first one corresponds to a short time where the slope coefficient on the log–log graph is usually close to one; this region defines the so-called short-time diffusion coefficient. The second one is called the diffusion slowdown area where the slope coefficient is less than unity (anomalous diffusion). The third one determines the long-time diffusion, where the normal diffusion behavior is recovered with the slope coefficient close to one (assuming the system is below the percolation threshold) or the system is subdiffusive with the slope coefficient less than 1.0, which is an expression of approaching the percolation threshold. The site percolation threshold for the triangular lattice is 0.5 [13]. In Figure 4a,b, it can be observed that the curves corresponding to the concentration of obstacles between 0.02 and 0.12 show a very small deviation from the Fickian diffusion; only a very weak deviation in the diffusive behavior and slowing down is visible for both solvent molecules and dimers. Outside this region, the diffusion remains Fickian. For curves where the obstacle concentration is between 0.14 and 0.20, it can be observed that in the short-time region, the diffusion is still Fickian; in the slow-down region, the deviation from the normal diffusion becomes more pronounced; and for long times, there is a tendency to return to the normal diffusion (not all curves have been included in the figure for clarity reasons). For concentration obstacles higher than 0.20 and lower than 0.28, different dynamic behaviors can be observed for solvent molecules and dimers. While the behavior of the solvent molecule is similar to the cases with less obstacle content in the system, the dimers show distinct subdiffusion behavior for long times (this type of behavior is represented by the curve corresponding to the obstacle concentration of 0.24). For obstacle concentrations higher than 0.28, only two regimes are observed, namely the short-time regime, where diffusion is normal, and the second one with subdiffusive motion (with α < 1 clearly visible) for both the solvent and dimers.

The parameter that is often used to monitor the deviations from Fickian diffusion behavior is the so-called non-Gaussian parameter *α*_2_(*t*). This parameter allows us to estimate approximate changes in the nature of diffusion observed in Figure 4a,b with the increase in the concentration of obstacles. This parameter is defined as follows:

(6) $$\alpha_{2}\left(t\right)=\frac{\langle ∆r^{4}(t)\rangle }{2{\langle ∆r^{2}(t)\rangle }^{2}}-1$$

**Figure 5:**
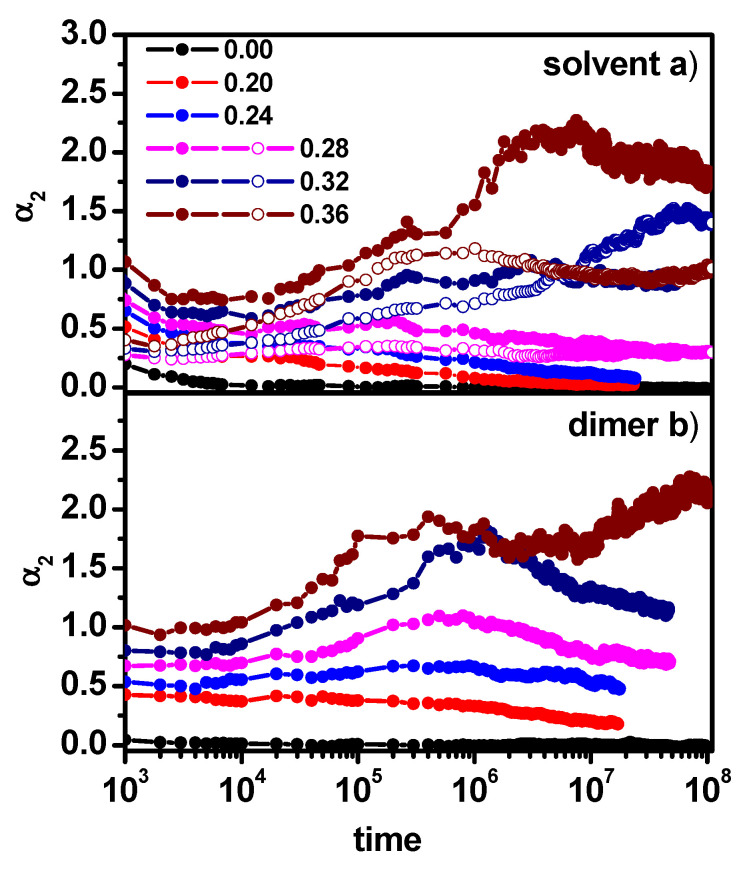
The non-Gaussian parameter *α*_2_(*t*) for solvent (**a**) and dimers (**b**) as a function of time. Solvent molecules in the system solvent + obstacles + dimers (solid symbols) and in the system solvent + obstacles (open symbols). The concentrations of obstacles are given in the inset. The concentration of dimers is 0.5.

This parameter shows how the distribution of object displacement deviates from the Gaussian distribution and thus, it is a measure of the dynamic heterogeneity of the studied system. The non-Gaussian parameter is close to zero when normal (Fickian) diffusion takes place and shows values greater than zero in cases of non-Fickian diffusion. In Figure 5a,b, changes in the non-Gaussian parameter with time are shown for some concentrations of obstacles and for the concentration of dimers at 0.5. It can be observed that the *α*_2_(*t*) behavior exactly reflects the diffusion behavior presented in Figure 4a,b for the solvent; for obstacle concentrations below 0.20, it does not reach too high values, and there is a clear tendency to return to normal diffusion. In the range of obstacle concentration between 0.20 and 0.28, we observe an increase in the *α*_2_(*t*) parameter for long times in the case of dimers, while the solvent shows a tendency to return to normal diffusion. For concentrations of obstacles above 0.28, we can see a clear increase in the *α*_2_(*t*) parameter value for both the solvent and dimers; however, the deviation from the Fickian behavior in the case of dimers seems to be greater, which is consistent with the results observed in Figure 4. Figure 5a shows a comparison of the non-Gaussian parameter for the solvent for a given concentration of obstacles in systems with and without dimers. In this case, it is visible that the presence of dimers has an effect on the solvent dynamics, which is manifested by higher values of the *α*_2_(*t*) parameter in the cases considered.

In order to complete the picture of the dynamics of dimers and solvent molecules, we study the changes in the cos(*φ*) autocorrelation function for the dimers and the position autocorrelation function for the solvent *ρ*(*t*).

**Figure 6:**
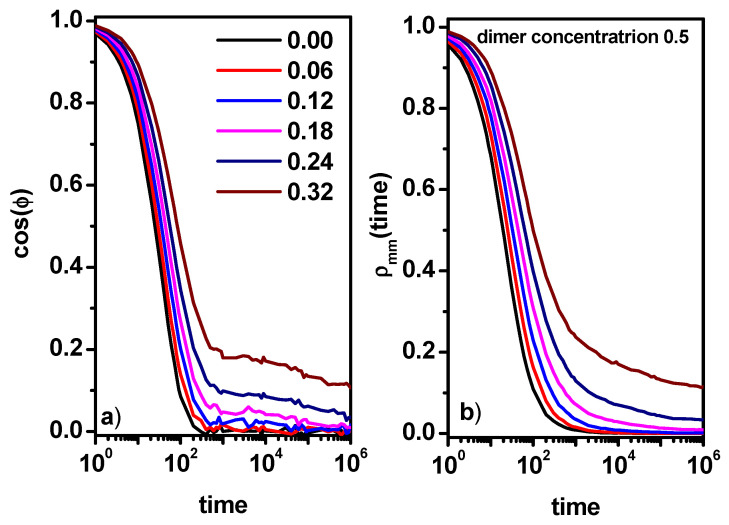
The end-to-end dimer autocorrelation function cos(*φ*) (**a**) and the solvent position autocorrelation function *ρ*(*t*) (**b**). The concentrations of obstacles are given in the inset. The concentration of dimers is 0.5.

Figure 6a,b presents the end-to-end dimer autocorrelation function and the solvent position autocorrelation function *ρ*(*t*) as a function of time. In Figure 6a, it can be observed that in the case of low concentrations of obstacles, the rotation of dimers does not show large deviations in relation to the results concerning the system without obstacles (Figure 2b); however, a small tail is visible, which would indicate processes related to the interactions of dimers with obstacles. For higher concentrations of obstacles, two processes can be clearly distinguished, a process in which a fast relaxation of the dimer takes place and a very slow relaxation related to the presence of obstacles. These two processes can be related to the dynamic behavior observed in the case of the MSD (Figure 4a). The fast relaxation corresponds to the short-time diffusion, while the rest of the curve corresponds to the behavior following it. Similar behavior for short times is observed in the case of the solvent autocorrelation function *ρ*(*t*) in Figure 6b. For long times, the relaxation process corresponding to longer times is not visible. Instead of this, it is visible that the autocorrelation function has approached a constant value, which indicates the caging of some solvent molecules. The higher value of the position autocorrelation function indicates more obstacles in the system, which corresponds well with the end-to-end dimer autocorrelation function (Figure 6a). When discussing the dynamics of dimers and solvent molecules for a constant dimer concentration of 0.5, it was worth noting that in a certain range of obstacle concentrations, the nature of the dynamics of solvent molecules and dimers differs significantly, i.e., dimers show a distinct subdiffusion behavior in the long term, while the solvent diffusion remains Fickian. A question has to be asked about the influence of the concentration of dimers on the difference in the solvent and dimer mobility.

**Figure 7:**
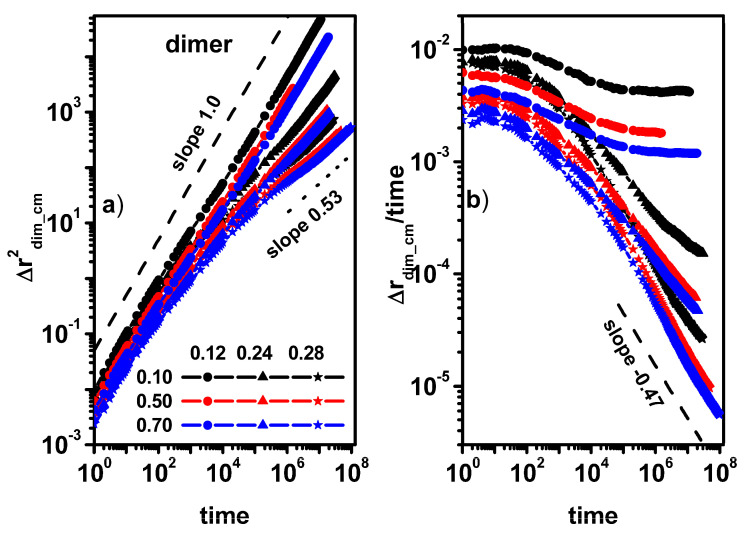
The MSD of dimers as a function of time (**a**) and MSD/time as a function of time (**b**). The concentrations of dimers (0.10, 0.50, and 0.70) and obstacles (0.12, 0.24, and 0.28) are given in the inset.

Figure 7a,b shows the MSD of dimers for different concentrations of dimers. It can be seen that in the case of low concentrations of obstacles, the effect of changing the dimer concentration does not affect the nature of the dimer movement, i.e., diffusion in the entire range of observed time (outside the diffusion slowdown region) remains Fickian. For higher dimer concentrations, in all cases, we observe (with the exception of the case of a dimer concentration of 0.1 and an obstacle concentration of 0.24, where a tendency to return to normal diffusion is visible) a deviation from normal diffusion.

**Figure 8:**
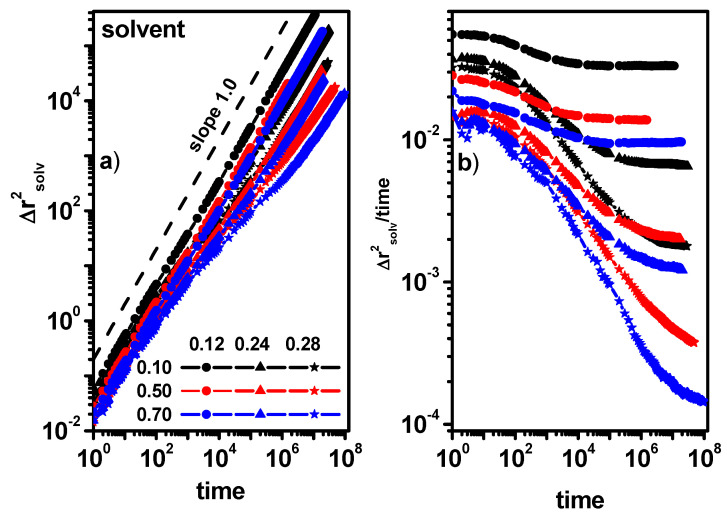
The MSD of solvent molecules as a function of time (**a**) and MSD/time as a function of time (**b**). The concentrations of dimers (0.10, 0.50, and 0.70) and obstacles (0.12, 0.24, and 0.28) are given in the inset.

Figure 8 shows the MSD and MSD/t for the solvent molecules for the same dimer and obstacle concentrations as in Figure 7. The situation is different for the solvent molecule when compared to dimers; in all the presented cases, the diffusion outside the transitional region of diffusion slowdown shows a Fickian character or approaches it for long times. It should be emphasized that such a situation is also observed when the concentration of obstacles is 0.28 and the concentration of dimers is 0.70, i.e., the concentration of the solvent in the system is 0.02. The movement of dimers and solvent molecules is closely correlated and despite this, the nature of the movement of dimers and solvent shows different properties.

The results presented above in Figure 7 and Figure 8 can be confirmed by the behavior of the non-Gaussian parameter *α*_2_(*t*).

**Figure 9:**
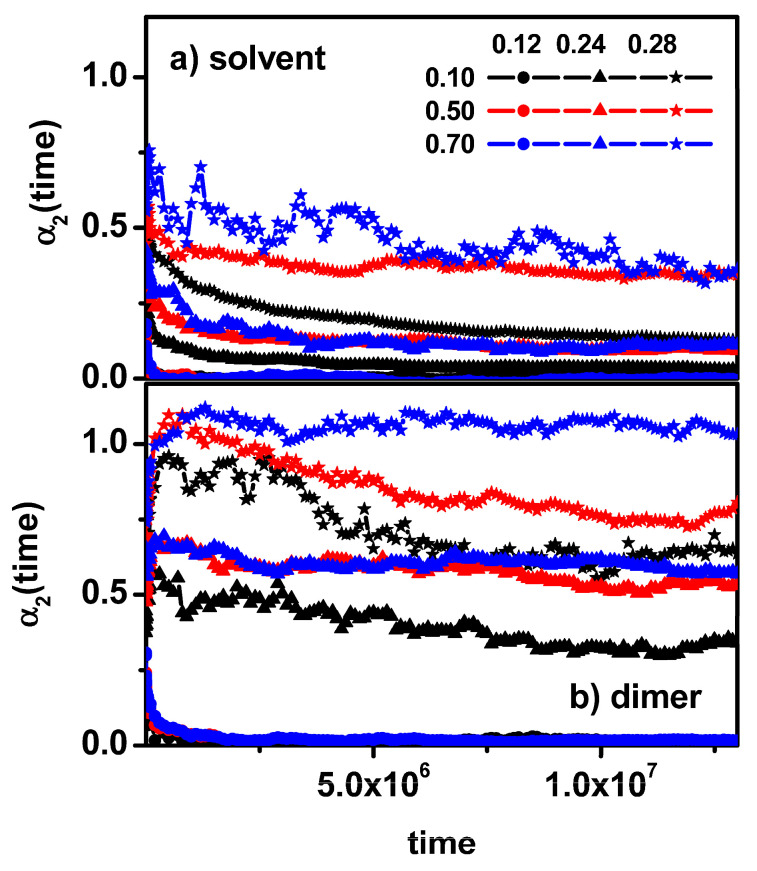
The non-Gaussian parameter α_2_(*t*) as a function of time for solvent molecules (**a**) and dimers (**b**). The concentrations of dimers (0.10, 0.50, and 0.70) and obstacles (0.12, 0.24, and 0.28) are given in the inset.

Figure 9 presents the changes in the non-Gaussian parameter of dimers and solvent molecules. It can be observed that the deviation from normal diffusion in the case of low obstacle concentration is practically not observed for dimers and solvent molecules. In the remaining cases, the observed values of the α_2_(*t*) parameter for dimers significantly exceed the values of the α_2_(*t*) parameter observed for the solvent, which is consistent with the results presented in Figure 7 and Figure 8.

**Figure 10:**
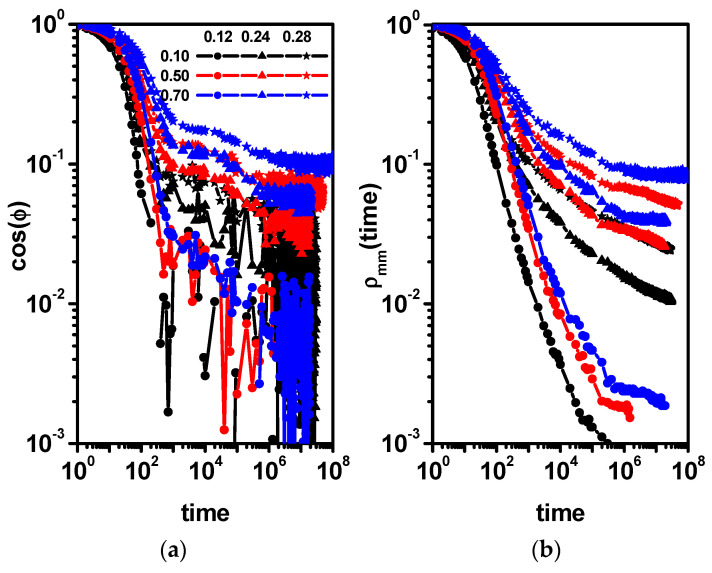
The end-to-end dimer autocorrelation function cos(*φ*) (**a**) and the solvent position autocorrelation function *ρ*(*t*) (**b**). The concentrations of dimers (0.10, 0.50, and 0.70) and obstacles (0.12, 0.24, and 0.28) are given in the inset.

Figure 10 shows the end-to-end dimer autocorrelation function cos(*φ*) and the solvent position correlation function *ρ*(*t*). The plots are drawn on a double logarithmic scale to observe the long-time behavior of these functions. The results presented in Figure 10a indicate that with the increase in the concentration of dimers and obstacles, the relaxation time corresponding to the short-time diffusion systematically increases. At higher concentrations of obstacles and dimers, dimer relaxation processes appear. They correspond to longer times and are related to the correlation between the motion of a dimer and neighboring dimers and the presence of obstacles. For high concentrations of obstacles, a steady but small decrease in the autocorrelation function can be seen, though for the longest times, we probably see the beginning of a plateau. This indicates the possibility of the caging of some dimers by the obstacles. It is difficult to quantitatively relate changes in the nature of the MSD (diffusion vs. subdiffusion) to the behavior of the two functions shown in Figure 10. In the case of the solvent (Figure 10b), the picture is similar. The value of the autocorrelation function of the position of the solvent for long times also decreases, although this decrease in value is increasingly smaller. Only for high dimer concentrations does the function approach a constant value, indicating the caging of some solvent molecules. Dimer relaxation and solvent relaxation are, of course, different phenomena, but as a result of the close correlation in motions and due to the presence of obstacles, the curves look similar because as the number of obstacles increases, the number of trapped solvent and dimer molecules increases, hindering the motion of both as well as other solvent and dimer molecules. In this case, both translational and rotational motion is involved; moreover, in the correlation of the movement of dimers with other elements, there is the source of their earlier entry into the critical region (earlier observation of anomalous diffusion). In Figure 6 and Figure 10, we can see that the position relaxation curves are qualitatively similar, and we observe one relaxation process related to the change in object position and, in the case of increasing concentrations of obstacles, the tail observed for long times, which is a consequence of the increasing number of objects trapped by obstacles. On the other hand, in the case of the rotation autocorrelation function for low concentrations of obstacles, we observe one relaxation time associated with the rotation of a single dimer, and with increasing concentrations of obstacles, a second relaxation process begins to appear associated with correlations of its movement with other elements that make up the system, i.e., obstacles, solvent molecules, and other dimers. In the case of a high concentration of obstacles, in addition to these two processes, a hindering of dimer rotation is also evident, which manifests itself in the form of a plateau. Some solvent molecules are trapped in cages formed by the obstacles during the entire simulation run. For systems with the highest concentration of obstacles, that is, *c* = 0.28, in this situation, it is about 9% of solvent molecules. Not taking these molecules into account reduces the plateau value of the autocorrelation function in Figure 10b from about 0.5 to about 0.4 but not to zero. This means that a considerable fraction of the solvent molecules was additionally caged for long times. This situation was already discussed in Ref. [64]. Trapped molecules do not significantly impact the long-time MSD. Trapped molecules maintain a constant MSD over time, while the MSD of diffusive particles increases linearly. In a weighted MSD that accounts for both trapped and diffusive particles, the linear term will eventually dominate the constant term as time approaches infinity. A similar effect must be expected for dimers, but one has to remember that dimers for this concentration already exhibit subdiffusive behavior.

MSD curves allow for an easy determination of parameters that characterize a diffusion behavior of dimers and solvent molecules for a given composition of the system. One can perform this by finding the α exponent determined for long times according to Equation (3) and determining the short-time diffusion coefficient *D_SH_* (*t* < 10^2^) and the diffusion coefficient corresponding to long-time *D_L_* (*t* > 10^7^).

**Figure 11:**
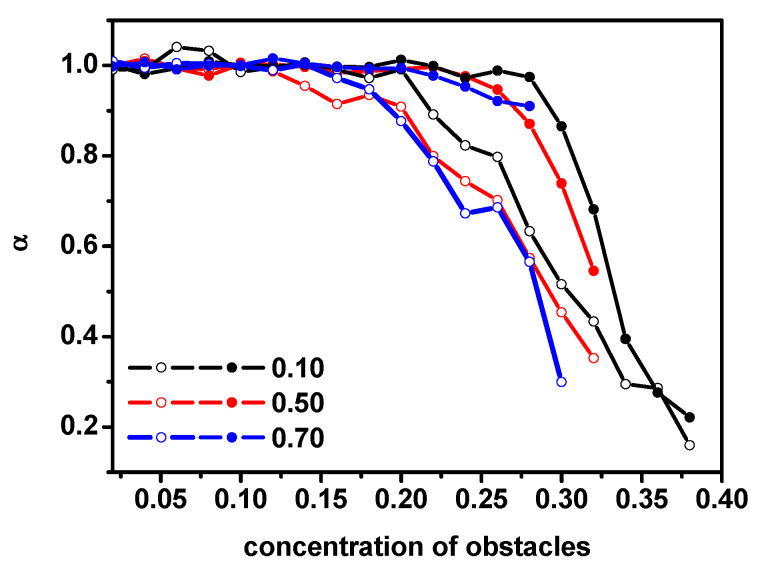
The exponent α from Equation (3) as a function of the obstacle concentration for solvent molecules (solid symbols) and dimers (open symbols).

Figure 11 presents the changes in the exponent *α* with the concentration of obstacles for solvent molecules and dimers. A character of the diffusion in long time can be clearly visible here: α = 1 (normal diffusion) can be found for concentrations below the percolation threshold and α < 1 (subdiffusion) when we approach this critical point. Near the percolation threshold, the decrease in exponent α is observed in both cases (dimers and solvent molecules); however, the presented results indicate that the percolation threshold for dimers is observed for lower obstacle concentrations than in the case of the solvent. Moreover, with the increase in the dimer concentration, both dimers and solvents enter the critical range earlier, i.e., for lower obstacle concentrations.

**Figure 12:**
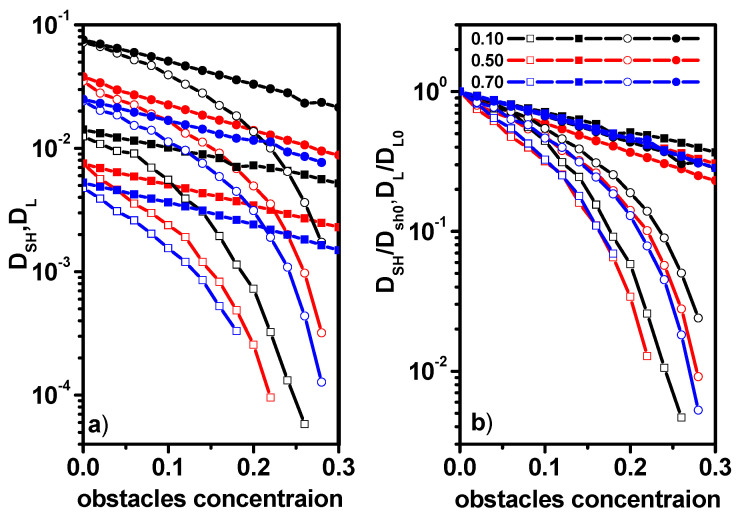
Short-time *D_SH_* (solid symbols) and long-time *D_L2_* (open symbols) diffusion coefficients (**a**) and relative diffusion coefficients *D_SH_*/*D_SH0_* and *D_L_*/*D_L0_* (**b**) as a function of obstacle concentration. The case of dimers (squares) and the solvent (circles). The concentrations dimers (0.10, 0.50, and 0.70) are given in the inset.

Figure 12a shows the short-time diffusion coefficient for dimers and solvent molecules and the long-time diffusion coefficient for dimers and the solvent for various concentrations of dimers and obstacles. Figure 6b presents the relative short-time *D_SH_*/*D_SH0_* and long-time diffusion coefficients *D_L_*/*D_L_*_0_. *D_SH_*_0_ and *D_L_*_0_ correspond to the diffusion coefficient of a system in which there are no obstacles at short-time and long-time cases, respectively. The diffusion coefficients for long times were determined only when the diffusion remained normal, i.e., when the α exponent varied within the range of 0.9 < α < 1. One can see that short-time diffusion coefficient in cases of dimers as well as the solvent decreases with the obstacle concentration exponentially: *D_SH_* = *D*_0_exp(−*ac*), where *c* is the concentration of obstacles. It seems that a similar relationship has been found experimentally and by molecular dynamics simulations but for systems without obstacles [10]. In Ref. [10], the ratio of diffusion coefficients was determined for diffusion in the short time only. Our results concerning diffusion in the long time are new. Unfortunately, there are no experimental data for comparison, while as far as simulations are concerned, a direct comparison with the results of molecular dynamics simulations is not likely to be possible, since the time scale as well as the density are unattainable for this method. The long-time diffusion coefficient in both cases decreases much faster with the increase in obstacles and the dependency on time is more complex. It should be noted that while the absolute values of the diffusion coefficients differ significantly (Figure 12a), the relative values presented in Figure 12b do not show much difference, especially in the case of short-time diffusion in both cases of dimers and the solvent.

**Figure 13:**
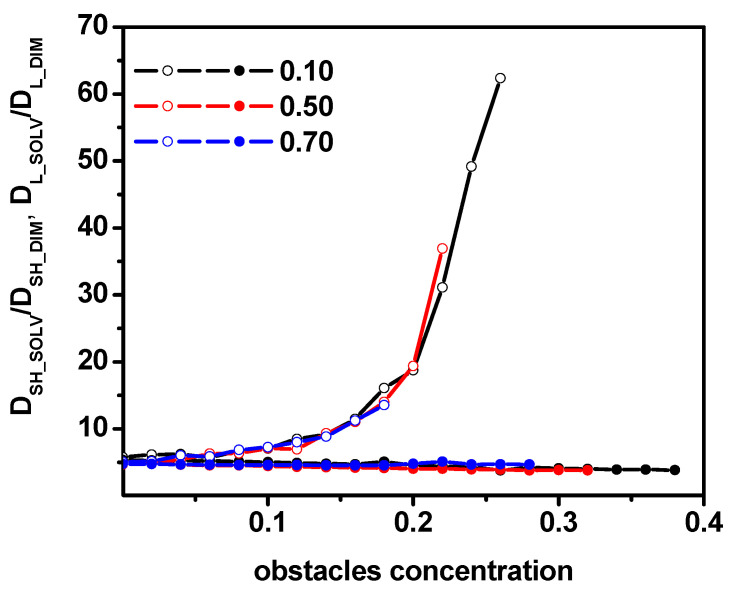
The ratios of diffusion coefficients for the solvent and dimers, D_SH-SOLV_/D_SH-DIM_ (solid symbols) and D_L-SOLV_/D_L-DIM_ (open symbols). The dimer concentrations (0.10, 0.50, and 0.70) are given in the inset.

We have characterized the dynamics of both species, i.e., solvent molecules and dimers, over a wide interval of the concentration of obstacles for various concentrations of dimers. In order to quantify the difference in the diffusivities of solvent particles and dimers, we consider the ratio of the short-time diffusion coefficient of solvent molecules and dimers D_SH-SOLV_/D_SH-DIM_ and also the ratio of the long-time diffusion coefficient of solvent molecules and dimers D_L-SOLV_/D_L-DIM_. These ratios are explicitly shown in Figure 13 as a function of the obstacle concentration for various concentrations of dimers. For the short-time regime, this ratio is practically independent (there is a slight downward trend with increasing obstacle concentration) of both the concentration of obstacles and concentration of dimers over the entire range of obstacle concentrations, where diffusion remains normal. This result is consistent with the experimental and molecular dynamics simulation results [10]. The ratio obtained by us is significantly (almost an order of magnitude) larger than the results in Ref. [10], but one must keep in mind the obstacles and the high density in our model. No less interesting is the result describing the ratio of diffusion coefficients in the case of long times. It can be observed that initially for small concentrations of obstacles, *c* < 0.1 remains approximately constant, then in the range between 0.1 and 0.2, a weak increase is visible and for concentrations greater than 0.2, the increase becomes rapid. Moreover, this ratio remains the same regardless of the concentration of dimers. The independence of D_SH-SOLV_/D_SH-DIM_ and D_L-SOLV_/D_L-DIM_ ratios from dimer concentration can be justified by the procedure proposed in Ref. [74]. It was shown there that the diffusion coefficients can be arranged along a single curve by introducing reduced values, that is, divided by the coupling parameter *Γ* (dimensionless interaction ratio). Carrying out a similar procedure in our system leads to independence of the considered ratios from the concentration of dimers (although the parameter *Γ*, which depends on the concentration of the solvent, dimers, and obstacles, cannot be determined in our case).

## 4. Conclusions

A coarse-grained model of a two-dimensional colloidal suspension was designed. A lattice approximation was introduced into this athermal model, and its properties were determined by Monte Carlo simulations. Due to the inclusion of explicit liquid molecules, which implies a very high density, a simulation algorithm called dynamic lattice liquid was used. A system consisting of solvent (monomer) molecules, dimer molecules, and immobile and impenetrable obstacles that introduce additional heterogeneity into the system was studied.

It is shown that there is a range of hindrance concentrations in which different diffusion characteristics are observed for dimers and solvents. It has been shown that the ratio of diffusion coefficients for short times exhibits the same properties as in previous work [10]. Thus, it is shown that both changing the concentration of dimers and obstacles practically does not change this ratio. It is also important to note the behavior of diffusion coefficients for long times. In a system, it is possible to define areas of concentration of the individual components (solvent, dimers, and obstacles) where the nature of the movement of dimers and the solvent is different. As for the ratios of diffusion coefficients of solvent molecules and dimers for short times, it can be seen that they do not depend on the obstacle density (and even systems without obstacles behave similarly). For longer times, the ratio of the two diffusion coefficients increases but is independent of the dimer concentration.

## Data Availability

The dataset is available upon request from the authors.
